# Using Whole Genome Analysis to Examine Recombination across Diverse Sequence Types of *Staphylococcus aureus*

**DOI:** 10.1371/journal.pone.0130955

**Published:** 2015-07-10

**Authors:** Elizabeth M. Driebe, Jason W. Sahl, Chandler Roe, Jolene R. Bowers, James M. Schupp, John D. Gillece, Erin Kelley, Lance B. Price, Talima R. Pearson, Crystal M. Hepp, Pius M. Brzoska, Craig A. Cummings, Manohar R. Furtado, Paal S. Andersen, Marc Stegger, David M. Engelthaler, Paul S. Keim

**Affiliations:** 1 Pathogen Genomics Division, The Translational Genomics Research Institute, Flagstaff, Arizona, United States of America; 2 Center for Microbial Genetics and Genomics, Northern Arizona University, Flagstaff, Arizona, United States of America; 3 Thermo Fisher Scientific, South San Francisco, California, United States of America; 4 Microbiology and Infection Control, Statens Serum Institut, Copenhagen, Denmark; Leibniz-Institute DSMZ, GERMANY

## Abstract

*Staphylococcus aureus* is an important clinical pathogen worldwide and understanding this organism's phylogeny and, in particular, the role of recombination, is important both to understand the overall spread of virulent lineages and to characterize outbreaks. To further elucidate the phylogeny of *S*. *aureus*, 35 diverse strains were sequenced using whole genome sequencing. In addition, 29 publicly available whole genome sequences were included to create a single nucleotide polymorphism (SNP)-based phylogenetic tree encompassing 11 distinct lineages. All strains of a particular sequence type fell into the same clade with clear groupings of the major clonal complexes of CC8, CC5, CC30, CC45 and CC1. Using a novel analysis method, we plotted the homoplasy density and SNP density across the whole genome and found evidence of recombination throughout the entire chromosome, but when we examined individual clonal lineages we found very little recombination. However, when we analyzed three branches of multiple lineages, we saw intermediate and differing levels of recombination between them. These data demonstrate that in *S*. *aureus*, recombination occurs across major lineages that subsequently expand in a clonal manner. Estimated mutation rates for the CC8 and CC5 lineages were different from each other. While the CC8 lineage rate was similar to previous studies, the CC5 lineage was 100-fold greater. Fifty known virulence genes were screened in all genomes *in silico* to determine their distribution across major clades. Thirty-three genes were present variably across clades, most of which were not constrained by ancestry, indicating horizontal gene transfer or gene loss.

## Introduction

*Staphylococcus aureus*, a major human pathogen that can cause skin and soft tissue infections as well as fatal disease due to pneumonia, endocarditis and osteomyelitis, continues to be of concern in both hospital and community settings, especially given the high rates of antibiotic resistance [1]. Resistance to beta-lactams, including methicillin, in *S*. *aureus* was first detected in 1961 [2] and methicillin-resistant *S*. *aureus* (MRSA) continues to cause significant disease and mortality today. In 2005, more deaths occurred from MRSA infections in the US than from AIDS [3]. Understanding the evolution of this pathogen is important, but there have been few analyses employing whole genome sequencing, looking at the overall relationships among different clonal groups of *S*. *aureus*.

There have been several whole genome studies in *S*. *aureus* focused on a single sequence type. Harris *et al*. 2010 [4] sequenced 63 isolates of sequence type (ST) 239, a globally disseminated healthcare-associated clone defined by multi-locus sequence typing (MLST). They presented data showing that CGS analysis revealed the global geographic structure for ST239 and demonstrated the possibility of using this technique to track transmission within a single hospital. In another clade-specific *S*. *aureus* study [5], next-generation sequencing was used to analyze 89 strains of clonal complex (CC) 398, a predominant livestock-associated *S*. *aureus* lineage. The resolution of whole genome SNPs allowed for the determination that CC398 likely has its origins as methicillin-sensitive *S*. *aureus* in humans, instead of animals, as was previously thought. A recent study of ST22 [6] allowed for phylogenetic reconstruction of an important European clone, EMRSA-15 and estimations of evolutionary rates and most recent common ancestor using Bayesian analysis.

Additionally, evidence for recombination has been detected in *S*. *aureus*, involving mobile genetic elements, homologous recombination as well as large-scale chromosomal replacements and our analysis supports recombination occurrences across the *S*. *aureus* genome. Monecke *et al*. [7] describe a high rate of genetic recombination from a microarray analysis of 3000 clinical and veterinary isolates; however they restricted their conclusions to the mobilome and did not intimate a recombination impact on the core genome. Other whole genome sequence analyses have been typically restricted to single lineages [4, 8–10] and have not been able to evaluate the level of recombination in *S*. *aureus* as a whole or compare across lineages. Early studies hinted that some recombination has occurred, but suggest that most *S*. *aureus* clonal variants have arisen by point mutation [11]. Takuno *et al*. [12] examined twelve published *S*. *aureus* genomes and detected homologous recombination at a relative rate of 0.6 to the mutation rate. However, the impact and possible differing levels of recombination across diverse sequence types in *S*. *aureus* remains undefined. A recent analysis across *S*. *aureus* lineages in a species-wide study found that widespread homologous recombination exists and mobile elements are associated with the strongest hotspots of recombination [13].

Horizontal transfer of virulence genes can be a source of diversity and differing success among lineages. Virulence genes have been well studied in *S*. *aureus* [14, 15] and transfer of those genes has been intimated as a factor in the emergence of new strains of MRSA [16]. In addition to phylogenetic reconstruction, whole genome sequencing allows for the analysis of gene content across analyzed genomes [17].

In this study, we used whole genome sequencing of many of the dominant *S*. *aureus* lineages from the US and other regions of the world to: 1) infer genomic evolutionary patterns among lineages 2) examine recombination in *S*. *aureus* across and within clonal complexes 3) estimate mutation rates and the time to most recent common ancestor using Bayesian analysis and 4) determine the distribution of known virulence genes. We found few differences from MLST, a high level of recombination between, but not within clonal complexes, differing mutation rates between two common lineages and evidence of horizontal gene transfer or gene loss in most of the virulence genes interrogated. This study spotlights the evolution of two clinically important lineages (CC5 and CC8) and adds to our understanding of the underlying patterns of evolution in the species as a whole.

## Materials and Methods

### Bacterial Isolates

Whole genome sequencing (WGS) analysis was performed on a convenience set of a total of 35 isolates: thirty *S*. *aureus* strains using the Illumina Genome Analyzer_*IIx*_ (GA_*IIx*_) (Illumina Inc, San Diego, CA) and five *S*. *aureus* strains from MLST-derived Clonal Complex 8 (CC8) using the SOLiD system (Life Technologies Corp, Carlsbad, CA). Strains were selected based on diversity of previously performed PFGE typing to represent genetic diversity, although no PFGE was performed for this study. Twenty-four typed strains were acquired from the National Antimicrobial Resistance in *Staphylococcus* (NARSA) (http://www.narsa.net/content/home.jsp) repository where they were characterized by PFGE and SCC*mec* type. Four strains were selected from the ICARE study at Emory University [18] and were previously characterized only by PFGE, and one additional strain was provided by Laboratory Sciences of Arizona and was characterized by PFGE and the DiversiLab system (bioMérieux, Durham, NC) using rep-PCR. These strains included representatives of the majority of the PFGE types previously described in the United States [19]. Statens Serum Institute (SSI) in Denmark, also provided two strains from the dominant European community-acquired (CA) MRSA lineage, CC80. Strains from CC8 and CC5 as determined by MLST were heavily represented. One additional strain, N315, was re-sequenced as a confirmation of the analysis, but data from that strain is not included here. In addition, 29 publicly available whole genome sequences were used in the analysis (S1 Table). The majority (n = 52) of the strains were MRSA, although a few (n = 12) methicillin-sensitive *S*. *aureus* (MSSA), vancomycin-intermediate *S*. *aureus* (VISA) and vancomycin-resistant *S*. *aureus* (VRSA) were included.

### Bacterial culture and genomic DNA preparation

*S*. *aureus* strains were grown on blood agar (Hardy Diagnostics, Santa Maria, CA) for 24 hours. Genomic DNA was extracted using a Qiagen DNeasy Blood and Tissue Kit as per manufacturer’s instructions (Qiagen, Valencia, CA), with the addition of lysostaphin (Sigma-Aldrich, St Loius, MO) at 200ug/mL to the enzymatic lysis buffer and an incubation from 1 to 5 hours. Following extraction, quantification was performed on a NanoDrop 8000 (Thermo Scientific, Waltham, MA) with verification by agarose gel electrophoresis. Extracted DNA quantities were normalized to 10 to 15ng/uL in 200 uL, for final yields of 2 to 3 ug before library preparation.

### Indexed genomic library preparation and DNA sequencing

For isolates sequenced on the Life Technologies SOLiD platform, mate pair libraries were prepared using the manufacturer’s protocol (Life Technologies Corp). Briefly, sheared, methylated chromosomal DNA was prepared, adaptors were ligated, and the fragments were circularized. Digestion with EcoP151 resulted in 25-base pair tags separated by a distance corresponding to the initial library fragment size. After ligation of sequencing adaptors, library fragments were amplified and attached to beads by emulsion PCR, and the 25-base pair tags were sequenced.

For isolates sequenced on the Illumina GA_*IIx*_ (Illumina Inc) instrument, DNA samples were prepared for multiplexed, paired-end sequencing following the manufacturers protocol. For each isolate, 1–5μg dsDNA in 200μl was sheared and then purified using the QIAquick PCR Purification kit (Qiagen). Enzymatic processing of the DNA followed the guidelines as described in the Illumina protocol, with the exceptions of processing enzymes obtained from New England Biolabs (New England Biolabs, Ipswich, MA) and oligonucleotides and adaptors from Illumina (Illumina Inc). After ligation of the adaptors, the DNA was run on a 2% agarose gel for 2 hours; subsequently a gel slice containing 500–600 bp fragments of each DNA sample was isolated and purified using the QIAquick Gel Extraction kit (Qiagen). Individual libraries were quantified with qPCR on the ABI 7900HT (Life Technologies Corp.) using the Kapa Library Quantification Kit (Kapa Biosystems, Woburn, MA). Based on the individual library concentrations, equimolar pools of twelve *S*. *aureus* libraries were prepared at a concentration of at least 1nM. To ensure accurate loading onto the flowcell, the same quantification method was used to quantify the final pools. The pooled libraries were sequenced on the Illumina GA_*IIx*_ using “Genomic DNA sequencing primer V2” for 36 cycles. A 50 or 100bp read paired end run was used for all isolates. An average total of 1.76 million reads was obtained for each sample.

### MLST, *spa* type and virulence genes from whole genome sequencing

Reads from the Illumina GA_*IIx*_ were aligned against MLST variants for each of seven housekeeping genes using Lasergene’s Seqman NGEN version 2.2 software (Lasergene, Madison, WI), producing consensus sequence for each allele. For the SOLiD sequences, reference-based assemblies were performed using Life Technologies’ SOLiD system Analysis Pipeline Tool (Corona Lite). *De novo* assembly was performed for all genomes using the correction algorithm Spectral Alignment Error Correction followed by Velvet [20]. Assembled contigs were mapped to the MLST reference genes using MUMmer [21]. Consensus sequence for each of the genes was entered into the Locus and Allelic Profile Query on MLST.net to produce sequence types (STs). Additionally, MLST sequences from each query genome were concatenated and a maximum parsimony phylogenetic tree was generated using MEGA version 5 [22, 23] for comparison to the whole genome SNP tree. Clonal lineage associations were also determined by MLST mapping on www.spaserver.ridom.de or by comparison to similar *spa* types or *spa* repeat compositions to MLST analyzed isolates at the Danish National Staphylococcal Reference Laboratory at Statens Serum Institut, with subsequent grouping using eBURST at http://saureus.mlst.net/eburst/.

*spa* typing of all isolates was performed *in silico* from *de novo* assembled contigs using either Velvet 1.1 [20] or CLCbio’s Genomics Workbench 5.5 (CLCbio, Aarhus, Denmark). *spa* types were analyzed using Ridom StaphType (Ridom GmbH, Münster, Germany). Six strains were *spa* typed using conventional PCR and Sanger sequencing as previously described [24], five to confirm the *spa* types determined *in silico* and one from which a *spa* type could not be determined *in silico*.

Known *S*. *aureus* virulence genes were queried in the whole genome sequence data of all study strains. Panton-Valentine leukocidin S (*lukS-PV*) and Panton-Valentine leukocidin F (*lukF-PV*) (accession number AB186917.1) were used as alignment references for the sequencing reads in the Seqman NGEN V. 2.2 software. Presence of the genes was confirmed by visualization of read alignments across the entire 1,918bp region. To further assess the virulome, blast score ratio (BSR) analysis was used as previously described [17, 25] to query known virulence genes (S2 Table) and their homologs across all *S*. *aureus* strains. Briefly, TBLASTN [26] was used to align the peptide sequence of each virulence factor against all sequenced genomes, producing a query bit score. The score for each genome alignment was divided by the maximum bit score, produced by a self-alignment, to obtain the BSR for each virulence gene. The BSR value can range from 1.0 (100% similarity across 100% of the peptide) to 0 (no significant alignment). The Multi-experiment Viewer [27] was used to visualize the BSR values only across groups for which there were differing scores.

### SNP analysis

In order to determine the core genome SNPs, sequences were aligned against FPR3757, a closed USA300 *S*. *aureus* reference genome [28] for both the overall tree analysis and the CC8 strains. CC5 strains were aligned similarly against a closed *S*. *aureus* ST5 reference genome, N315 [29]. BFAST [30] was used for all alignments. Indels and reads mapping to multiple locations were removed from the final alignments. Each alignment was analyzed for single nucleotide polymorphisms (SNPs) using SolSNP (http://sourceforge.net/projects/solsnp/). Only loci that had a minimum coverage of 10X and the base variant was present in greater than 90% of the calls, were included in the final analysis. Additionally, duplicated regions were identified by a self-comparison of FPR3757 or N315 using MUMmer version 3.22 [21] and SNPs within these repetitive regions were removed. The 29 publicly available genomes were aligned using MUMmer/Nucmer. Results from SolSNP and the whole genome alignments were merged using a custom script. Importantly, only loci present in all strains were included and a matrix containing the core, orthologous SNPs was generated.

### Phylogenetic trees

To obtain phylogenetic trees, the matrix generated as described above was analyzed using maximum parsimony in MEGA version 5 [22, 23] and bootstrapped with 100 replicates. For groups with limited genetic variation and limited recombination such as a single species or within a single species, maximum parsimony is ideal for phylogenetic reconstruction [31] and provides for the use of homoplasy metrics that are the best indicators for phylogenetic accuracy in groups with little homoplasy [32] and can be used to identify recombined regions [5]. Additionally, phylogenetic trees were reconstructed using maximum likelihood for confirmation of results. The Hasegawa, Kishino, and Yano (HKY) model of nucleotide substitution[33] was incorporated, as this model had the lowest Bayesian Information Criterion score in a model comparison conducted in MEGA version 5 [23]. Strain relationships were largely robust to the choice of phylogenetic method. The first overall tree constructed included a publicly available whole genome sequence of *Staphylococcus epidermidis*; RP62A (accession number: NC_002976) as an out-group to determine the root of the tree. All subsequent trees, including the CC8 and CC5 trees, were rooted with the most basal taxa from that subgroup.

### SNP and homoplasy density

Recombined regions may contain a higher density of SNPs and the phylogenetic signal from those SNPs will conflict with the signal from clonally inherited regions. To determine the location and frequency of recombination, the SNP density was calculated using 1-kb non-overlapping regions that were taken from the reference genome, FPR3757. Each region was pulled from the SNP matrix generated as described above and parsimony informative (PI) sites were tabulated. To identify homoplastic SNPs, a maximum parsimony tree was inferred with PAUP v4.0b10, and a SNP was determined to be homoplastic if it had a CI value ≤ 0.5. All homoplastic SNPs were coordinated with the 1-kb fragments, and the ratio (homoplasy density) of homoplastic SNPs to all PI SNPs was calculated. The number of PI SNPs and the homoplasy density across each 1-kb window was plotted with Circos [34].

### Molecular clock analysis

To estimate evolutionary rates and divergence times of the different clonal complexes, we employed a Bayesian molecular clock method as implemented in the BEAST v1.8.0 software package [35]. First, SNPs with a Retention index (RI) value of < 0.5, as calculated by Paup v4a140, were manually removed from the multiple sequence alignment to filter recombinate regions from the data set. Similarly to the phylogenetic reconstruction using maximum likelihood, the HKY model of nucleotide substitution [33] was incorporated again here to describe nucleotide substitution patterns among taxa. Because only variable sites were included in this analysis, we implemented an ascertainment bias correction model, as done in Gray et al, [36]. Path sampling [37] and stepping stone [38] sampling marginal likelihood estimators were employed to determine the best fitting clock and demographic model combinations [39, 40] (S3 Table). These methods of statistical model selection indicated that the combination of the uncorrelated lognormal molecular clock and the nonparametric Bayesian skygrid models best fit the data. The relaxed uncorrelated lognormal molecular clock model was used to infer the timescale and mutation rates while allowing for rate variation among lineages [41] with a gamma distribution prior on the mean clock rate (shape = 0.001, scale = 1000) and an exponential prior (mean = 1/3) on the standard deviation as recommended by Faria et al. [42]. Three independent Markov chain Monte Carlo (MCMC) chains were run for 500 million generations each, with parameters and trees drawn from the posterior every 50,000^th^ step. LogCombiner [35] was used to merge the samples from each chain, and the first 50% of each chain was discarded as burn-in. Visual trace inspection and calculation of effective sample sizes was conducted using Tracer [43] and confirmed MCMC mixing within and among chains. The posterior mean and 95% confidence intervals have been reported for the evolutionary rate and time to most recent common ancestor estimates.

## Results

Using next-generation sequencing technologies, we sequenced and mapped genome-wide core SNPs in 35 diverse strains, plus 29 publically available whole genome sequences of *S*. *aureus*. In addition, we used whole genome data to determine MLST and clonal complex assignments, SCC*mec* type, PVL status and *spa* type *in silico* for all strains, both publically available and newly sequenced (Table 1). Strains were chosen to represent much of the diversity seen in the US in *S*. *aureus*, but they do not represent the total diversity of the species. The number of reads per genome and statistics on the novel genome assemblies are presented in S4 Table and characteristics on all strains in the study are in S1 Table. All read data from the 35 strains sequenced in this study are deposited at NCBI in the short read archive under the BioProject accession number PRJNA214785.

**Table 1:** Genotype of *Staphylococcus aureus* isolates used in this study, same order as in phylogeny.

Strain	Isolate	SCC*mec* type	MLST	Clonal Complex	PVL +/-	*spa* by WGS
1	USA500-ICARE157	SCCMEC IV	ST8	CC8	PVL(+)	t008
2	USA300-ICARE392	SCCMEC IV	ST8	CC8	PVL(+)	t008
3	FPR3757	SCCMEC IV	ST8	CC8	PVL(+)	t008
4	USA300-OR-54	SCCMEC IV	ST8	CC8	PVL(+)	t2849
5	USA300-AZ-573	SCCMEC IV	ST8	CC8	PVL(+)	t008
6	USA300-0114	SCCMEC IV	ST8	CC8	PVL(+)	t008
7	USA300-ICARE043	SCCMEC IV	ST8	CC8	PVL(+)	t008
8	TCH1516	SCCMEC IV	ST8	CC8	PVL(+)	t622
9	USA300-CA-263	SCCMEC IV	ST8	CC8	PVL(+)	t008
10	IBERIAN-GA-356	SCCMEC IV	ST8	CC8	PVL(-)	t064
11	USA500-NRS385	SCCMEC IV	ST8	CC8	PVL(-)	t064
12	USA500-GA-355	SCCMEC IV	ST8	CC8	PVL(-)	t064
13	USA500-NY-177	SCCMEC IV	ST8	CC8	PVL(-)	t064
14	COL	SCCMEC I	ST250	CC8	PVL(-)	t008
15	Newman	none	ST254	CC8	PVL(-)	t008
16	NCTC 8325	none	ST8	CC8	PVL(+)	t211
17	TW20	SCCMEC III	ST239	CC8	PVL(+)	t037
18	JKD6008	SCCMEC III	ST239	CC8	PVL(-)	t037
19	T0131	SCCMEC III	ST239	CC8	PVL(-)	t030
20	MSSA476	none	ST1	CC1	PVL(-)	t607
21	MW2	SCCMEC IV	ST1	CC1	PVL(+)	t128
22	USA400-BAA1752	SCCMEC IV	ST1	CC1	PVL(+)	t125
23	USA900-20210	none	ST15	CC15	PVL(-)	t084
24	CC80-24329	SCCMEC IV	ST153	CC80	PVL(+)	t044
25	11819–97	SCCMEC IV	ST80	CC80	PVL(+)	t044
26	USA700-NRS386	SCCMEC IV	ST72	CC72	PVL(-)	t126
27	USA700-GA-442	SCCMEC IV	ST72	CC72	PVL(-)	t148
28	USA800-NRS387	SCCMEC IV	ST5	CC5	PVL(-)	t088
29	USA800-NY-313	SCCMEC IV	ST83	CC5	PVL(-)	t5576
30	ED98	none	ST5	CC5	PVL(-)	t002
31	Mu3	SCCMEC II	ST5	CC5	PVL(-)	t002
32	Mu50	SCCMEC II	ST5	CC5	PVL(-)	t002
33	N315	SCCMEC II	ST5	CC5	PVL(-)	t002
34	ECT-R2	pseudo-SCC	ST5	CC5	PVL(-)	t002
35	04–02981	SCCMEC II	ST225	CC5	PVL(-)	t003
36	JH9	SCCMEC II	ST105	CC5	PVL(-)	t002
37	JH1	SCCMEC II	ST105	CC5	PVL(-)	t002
38	USA100-OR-10	SCCMEC II	ST5	CC5	PVL(-)	t002
39	USA100-OR-293	SCCMEC II	ST5	CC5	PVL(-)	t2597
40	USA100-NY-76	SCCMEC II	ST5	CC5	PVL(-)	t002
41	USA100-NRS382	SCCMEC II	ST5	CC5	PVL(-)	t002
42	USA100-NY-54	SCCMEC II	ST105	CC5	PVL(-)	t002
43	USA100-CA-126	SCCMEC II	ST5	CC5	PVL(-)	t242
44	USA100-CA-248	SCCMEC II	ST5	CC5	PVL(-)	t242
45	JKD6159	SCCMEC IV	ST93	Singleton	PVL(-)	t202
46	ED133	none	ST133	CC133	PVL(-)	t2678
47	LGA251	SCCMEC XI	ST425	CC425	PVL(-)	t6300
48	RF122	none	ST151	CC705	PVL(+)	t529
49	MO13	SCCMEC V	ST59	CC59	PVL(+)	t437
50	USA1000-CA-629	SCCMEC V	ST87	CC59	PVL(-)	t216
51	USA1000-94318	SCCMEC IV	ST59	CC59	PVL(+)	t316
52	08BA02176	SCCMEC V	ST398	CC398	PVL(-)	t034
53	SO385	SCCMEC V	ST398	CC398	PVL(-)	t011
54	71193	none	ST398	CC398	PVL(-)	t571
55	USA1100-04031	none	ST30	CC30	PVL(+)	t019
56	MRSA252	SCCMEC II	ST36	CC30	PVL(-)	t018
57	USA200-NRS383	SCCMEC II	ST346	CC30	PVL(-)	t018
58	USA200-OR-131	SCCMEC II	ST36	CC30	PVL(-)	t012
59	USA600-BAA1754	SCCMEC IV	ST45	CC45	PVL(-)	t671
60	USA600-NY-315	SCCMEC II	ST45	CC45	PVL(-)	t132
61	USA600-CA-347	SCCMEC II	ST45	CC45	PVL(-)	t004
62	USA600-BAA1751	SCCMEC II	ST45	CC45	PVL(-)	t266
63	USA600-NRS22	SCCMEC II	ST45	CC45	PVL(-)	t266
64	MSHR1132*	SCCMEC IV	ST1850	CC75	PVL(-)	novel**

*not included in final tree.

**repeats 259-31-17-17-17-22-17-17-23-17-22.

### Phylogenetic trees and Recombination

**Fig 1:**
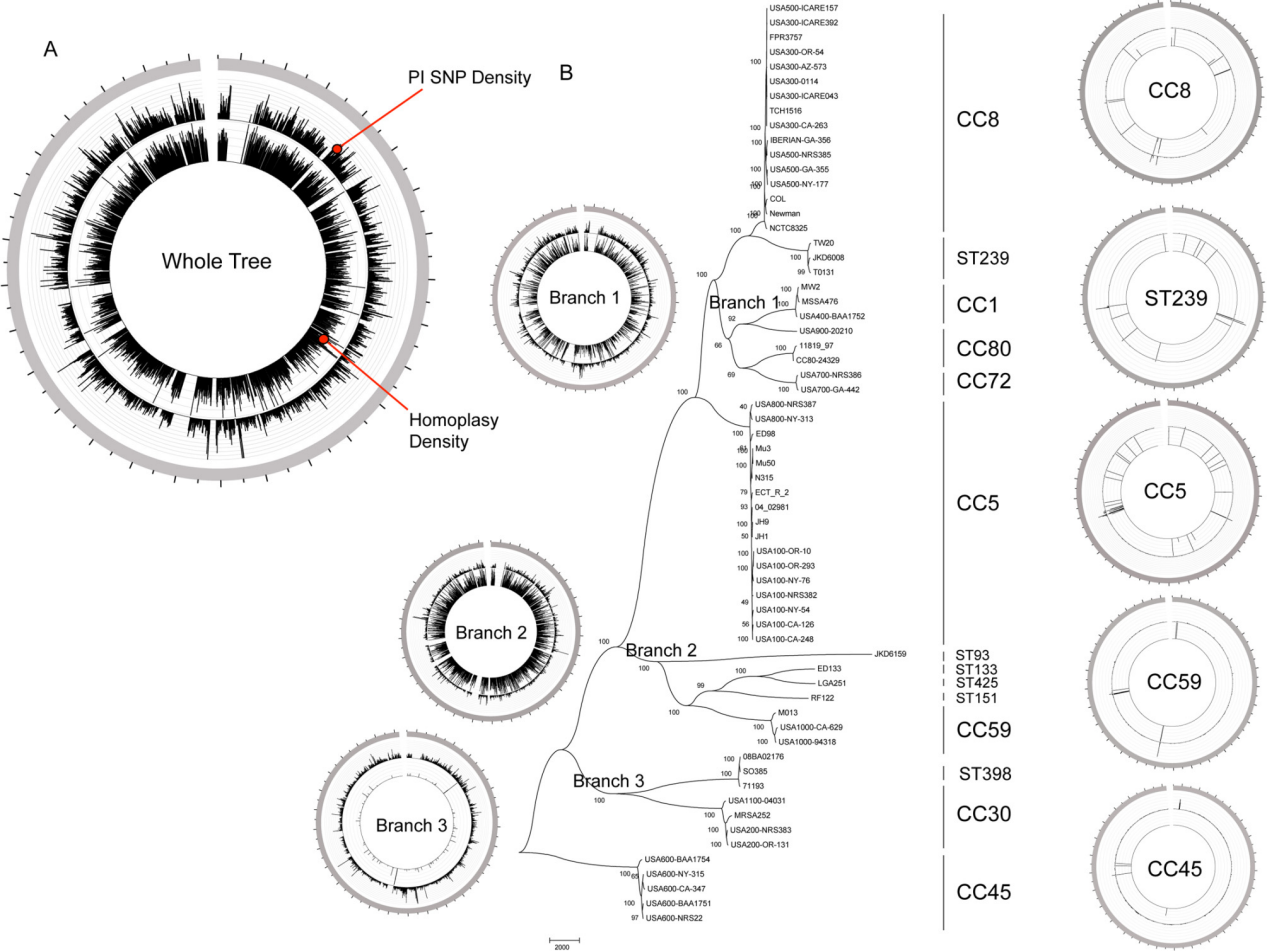
Evolutionary relationships of *S*. *aureus* clones found in the US and abroad. (A) Circular map indicating homoplasy and SNP density in all taxa. Outer grey circle indicates the core reference chromosome beginning at the origin. The external track indicates the homoplasy density and the internal tack indicates parsimony informative (PI) SNPs for all strains analyzed showing dispersed homoplasy and PI SNP density. (B) Maximum-parsimony tree of 64 isolates of diverse *S*. *aureus* based on 80,836 SNPS, of which 57,236 were parsimony-informative, with a consistency index of 0.59. The numbers shown next to the branches represent the percentage of replicate trees where associated taxa cluster together based on 100 bootstrap replicates. The tree is rooted with the CC45 clade. Clonal complex or sequence types are indicated. Circular maps of homoplasy and SNP density for four of the clonal complexes and one sequence type (ST239) are located to the right of the tree and those for five of the branches are located on the left of the tree.

The maximum parsimony phylogeny presented in Fig 1 was reconstructed using 80,836 SNPs identified in comparison to a *S*. *aureus* CC8 reference (FPR3757). Of the total SNPs, 57,236 were parsimony informative. The phylogeny had a consistency index (CI), excluding parsimony uninformative SNPs, of 0.59 which indicates a moderate level of homoplasy. The majority of bootstrap support values (41/52) were greater than 90%, indicating strong confidence in the groupings. The phylogenetic tree was rooted by the CC45 clade, which was determined to be most basal from a tree rooted with a near-neighbor, *S*. *epidermidis* (S1 Fig). This original tree included the northern Australian CC75 isolate, MSHR1132 (accession number: FR821777) however; this genome was removed for subsequent trees because of its large patristic distance from the other strains included in this study. MSHR1132 is more than 17,500 SNPs distant from the nearest *S*. *aureus* strain and nearly 40,000 SNPs distant from *S*. *epidermidis* and clearly represents a unique, basal clade as has previously been reported [7, 44]. Strains TW20, T0131 and JKD6008 are closely related to the CC8 clade and share a recent common ancestor. These strains belong to the MLST sequence type ST239, a hybrid known to have resulted from a large-scale recombination event whereby an ST8 strain acquired an approximate 635 kb region from an ST30 donor [45, 46], which is consistent with their placement on the overall phylogenetic tree. Although ST239 belongs to CC8 using MLST, we analyzed those strains separately from other CC8 strains, as they are quite distant from the other CC8’s (3943 SNPs) and clearly represent a distinct clade.

Homoplasy density within closely related taxa can indicate recombination and PI SNP density can highlight regions of recombination from outside groups. When plotted across the entire chromosome in a circular schematic diagram to identify recombined regions from both closely related taxa and outside groups, the homoplasy and PI SNP density patterns of the core genome SNPs (Fig 1A) showed many more recombined regions than when subclades were analyzed in isolation (Fig 1B). In the homoplasy density plot of the diverse CC phylogeny (Fig 1A, inner circle), homoplasy is scattered throughout the genome, indicating multiple recombination events dispersed across the genome over the evolutionary history of the species. The PI SNP density of the whole tree (Fig 1A, outer circle) is dispersed across the whole genome with a few peaks indicating some regions where recombination has occurred. These data support the hypothesis that recombination, both from outside and closely related taxa, is common in the *S*. *aureus* genome when examined across diverse groups. Regions that were filtered from the analysis are visible in Fig 1A by a lack of SNP loci and most correspond to a genomic island or phage that is not present in all the strains analyzed.

For the individual clades, very little evidence of recombination is present when analyzed on their own (Fig 1B), as has been noted previously [4, 5, 8, 47]. Few PI SNPs and low homoplasy densities were observed when five single clades (CC8, ST239, CC5, CC59 and CC45) were analyzed. For example, the CC8 only analysis (top circular plot) demonstrated few regions of elevated homoplasy density, which is consistent with the high CI for this group of 0.99. There were 2 regions with elevated PI SNPs and both occurred in the same phage that was not included in the analysis of all taxa. In the CC5 only analysis (third circular plot from top), there is more evidence of recombination than in the CC8 analysis and this corresponds with the CI value of 0.76 calculated for this group. Elevated homplasy density is infrequent, but dispersed across the genome. The PI SNPs identified are clustered in a single region.

Data indicating recombination in the three deep branches of our phylogeny (Branch 1, Branch 2 and Branch 3 in Fig 1A) are also plotted across the whole genome. Each branch contains more than one clade and either seven or eight taxa that have similar branch lengths, with the exception of the branch leading to the ST93 strain, JKD6159. These three schematic circular diagrams indicate varying, but relatively high, levels of recombination present in each branch when compared to the individual clades. For example, the circular plot of Branch 1 shows some dispersed homoplasy density (inner circle) where the total homoplastic SNPs/total PI SNPs = 20.2%, compared to Branch 2, which shows much more (36.1%), and Branch 3, which shows very little (0.84%). The density of PI SNPs appears to be less variable across the three branches analyzed, but nonetheless highlights differing regions of high SNP densities. These data indicate varying recombination rates between branches and an intermediate level of recombination when compared to the whole tree and individual clades.

**Fig 2:**
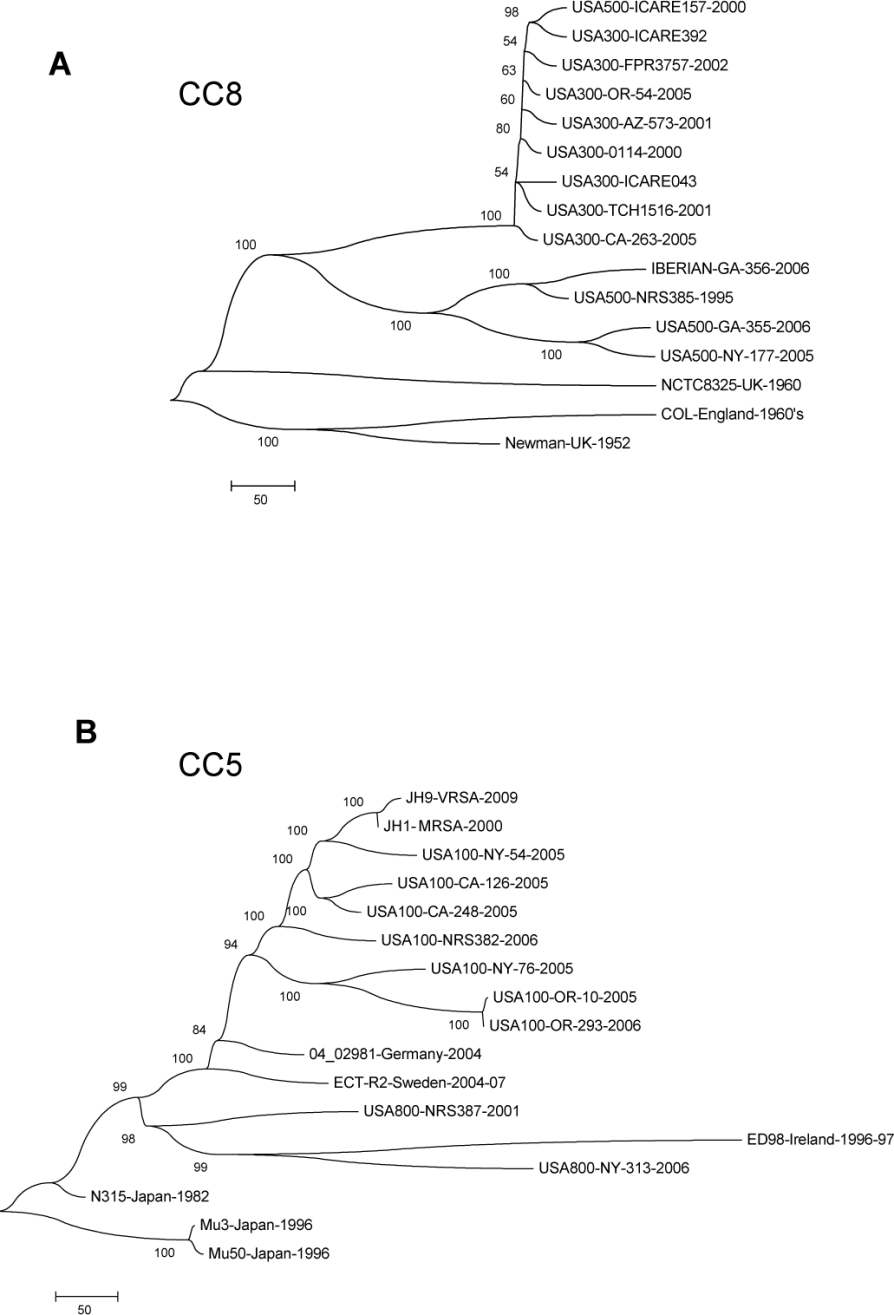
(A) Maximum-parsimony tree of the 16 strains belonging to the CC8 clade based on 1,717 SNPS (860 parsimony informative SNPs) with a consistency index of 0.99. (B) Maximum-parsimony tree of the 17 strains belonging to the CC5 clade based on 1,948 SNPS (632 parsimony informative SNPs) with a consistency index of 0.76.

The CC8 clade contained strains previously PFGE-typed as USA300 and USA500 (Fig 2A, CI = 0.99, 1,717 total SNPs), two clinically significant groups of MRSA in the US. Additionally, this group contained isolates typed as Iberian by PFGE. The taxa included in this group are consistent with previous characterizations of these PFGE types [48]. The USA300 MRSA isolates that were included in this clade are grouped in a single, tightly clustered sub-clade and all contained the PVL locus and SCC*mec* type IV (Table 1). The CC5 clade included strains with the PFGE types USA100 and USA800 (Fig 2B). This tree had a CI of 0.76 and was based on 1948 SNPs of which 632 were parsimony informative. Most of the USA100 isolates clustered on a single branch which may provide a good target for assay development for this group, previously defined by PFGE and representing the dominant strain associated with nosocomial infections in the US [49].

### Molecular clock analysis

Bayesian estimation of divergence times and nucleotide substitution rates on the *S*. *aureus* full data set, using a relaxed molecular clock, revealed that the time to the most recent common ancestor (TMRCA) was 16,673 (95% CI: 4484–35976) years with an estimated mean nucleotide substitution rate of 1.8 x 10^−5^ substitutions per nucleotide site per year (95% CI: 4.8 x 10^−6^–3.7 x 10^−5^). Other estimates for *S*. *aureus*, based on single lineages, have been estimated at 10^−6^ substitutions per nucleotide site per year [4, 6, 8], which falls within the distribution of our estimated mean rates determined from multiple lineages indicating similar rates are estimated from multiple and single lineages. Further, the same Bayesian analysis was applied to each of the two clades of strains, CC8 and CC5. For the CC8 clade, a similar nucleotide substitution rate to the overall data set, of 3.8 x 10^−5^ (95%CI: 1.8 x 10^−5^–8.9 x 10^−5^) was estimated. However for CC5, a value of 1.8 x 10^−3^ (95%CI: 1.1 x 10^−7^–4.5 x 10^−3^) substitutions per nucleotide site per year was estimated. Recent analysis of sterile site infection *S*. *aureus* isolates collected over time from the same patient, revealed a significantly higher microevolutionary rate in ST5 compared to ST8 strains[50]. For the CC5 group, a TMRCA of 190 (95% CI: 59–373) years versus an estimate of 2385 (95% CI: 588–4847) years for CC8 was determined, indicating that CC5 is a more recently emerged group. Additionally, the ST239 clade within CC8 was determined to have a TMRCA of 160 (95% CI: 34–332) years, where the 95% CI overlaps previous timing estimates for this clade [13, 36].

### *spa* type and PVL from whole genome sequencing

All isolates were *spa* typed *in silico* from the WGS data, which showed similar results to previous published *spa* types of the epidemic lineages [7]. Identical *spa* types did fall within a single clade, however each clade contained more than a single *spa* type, (Table 1, isolates are listed in the same order as the phylogeny in Fig 1. We performed traditional PCR and Sanger methods on five strains to confirm *spa* types found *in silico* and found that four matched between traditional and WGS methods. In the fifth strain, a single repeat of the same motif was missed where WGS determined the *spa* type as t004 (09-02-16-13-13-17-34-16-34) and the traditional method determined *spa* as t266 (09-02-16-13-13-13-17-34-16-34). However, both *spa* types are associated with the same clonal complex, CC45.

The PVL toxin genes *lukS*/*F*-*PV* were not only found in the USA300 isolates mentioned previously, other genomes also contained these genes that have been suspected as indicators of community acquired (CA) strains [51–53]. The two CC80 strains from Denmark also contained PVL, as well as two of the three strains from CC1 (USA400) and four additional strains, all within different clades.

#### Virulence Genes Screen

**Fig 3:**
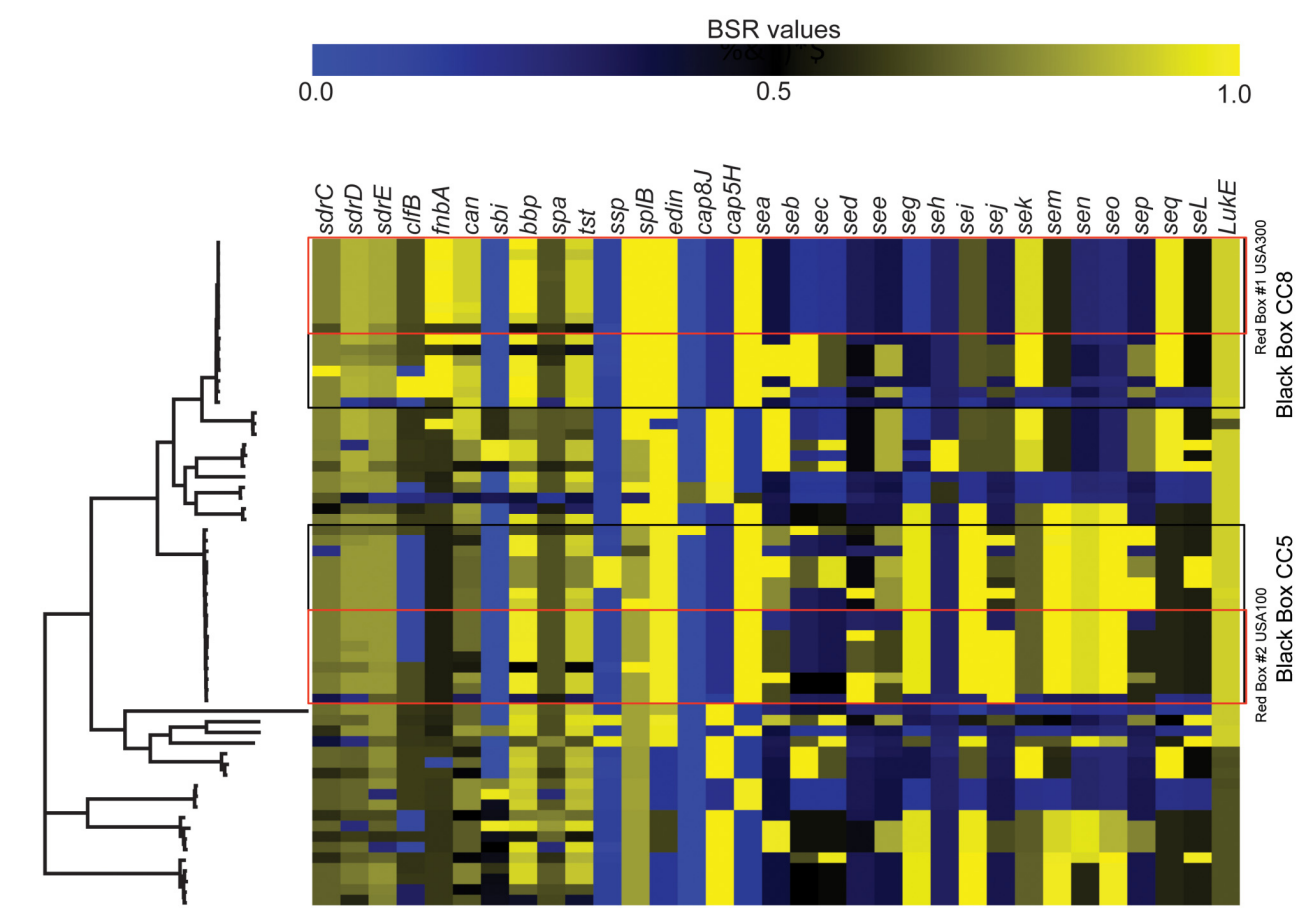
Heat map indicating the BSR values for virulence genes that varied across strains (33 of 50 screened). BSR values visualized with Multi-Experiment Viewer [27]. The left hand side of the figure contains the phylogeny as in Fig 1A. Clades are indicated with boxes; the black boxes indicated the CC5 and CC8 groups and the red boxes indicate the USA300 and USA100 groups. Gene names along with scale for heat map are indicated at the top of the heat map.

In addition to PVL, the major groups of known virulence genes were screened across all strains and results of only those genes that varied across clonal complexes are presented in Fig 3. The majority of the genes are chromosomal with a few exceptions (S2 Table). Strains are listed in the order that they grouped on the phylogenetic tree with the clonal complexes indicated. All raw BSR values for each marker screened in this study, including invariant genes, are listed in S5 Table.

The Staphylococcal enterotoxin genes exhibited distinct patterns, especially within CC8 that contains many strains of the CA-MRSA lineages. All USA300 strains sequenced contained the *sek* and *seq* genes, as well as an ortholog of *sei*, but not other enterotoxin genes, indicated by red box #1 in Fig 3. The majority of the USA500 strains collected in the last two decades, were positive for *sea*, *seb*, *see*, *sek* and *seq*. A different compliment of enterotoxin genes are present in the CC5 strains, including *seg*, *sei*, *sem*, *sen*, and *seo*. The USA100 strains, a subclade within the CC5 clade (Fig 3, red box 2), are the only group from which all members carry the plasmid-borne *sej* gene. This suggests that the presence of the sej may be important for the adaptive radiation of this predominantly hospital-associated (HA) lineage. CC45, which contains all of the USA600 strains, also has a unique set of enterotoxin genes, some of which are also present in the nearest lineage of CC30.

All genomes screened contained only one set of capsular polysaccharide genes that define either the CP5 or CP8 serotypes (*cap5H-J* and *cap8H-K*, respectively). Each clade contained only one serotype, either CP5 or CP8. The hemolysin genes were present in all genomes screened as were many of the leukocidins, other than PVL (S5 Table and Table 1). The fibrinogen-binding proteins screened were present in many of the isolates with *efb* being present in all. Nearly all three of the Ser-Asp rich fibrinogen-binding proteins, *sdr*C, D and E, were partially present in almost all genomes, which has not been shown previously. However, it is possible that these *sdr* genes are truncated in the assemblies due to repeat regions shared among the three genes, so the results could be due to bioinformatics analysis of short read data and not represent a true biological phenomenon.

## Discussion

The current study places well characterized clinical *S*. *aureus* strains, including many dominant US clonal groups, in relation to other known lineages based on WGS analysis. This high-resolution phylogenetic approach demonstrates the relationships among and between clonal groups of *S*. *aureus*. The mutational and recombinogenic differences among members within and among clades provide insights into the mutational patterns that have shaped the evolution of not only two clinically significant clades (CC8 and CC5), but also the entire species.

When comparing recombination across three levels of analysis (single clades, branches including more than one clade and the whole tree including representatives from fourteen different STs), we found recombination levels varied. In a novel analysis, we utilized homoplasy and PI SNP density as indicators of recombination within closely related taxa and from outside groups and showed clear recombination occurring across clonal lineages and clonal expansion within a single lineage. As expected, we demonstrated evidence of recombination dispersed throughout the genome with higher levels of recombination occurring across clonal groups than within. However, when analyzing the deep branches of our phylogenetic tree, we found that recombination was different for each of the three branches examined. Branch two had a homoplasy density similar to that of the overall tree indicating a very high level of recombination for this group, but differing from the other two branches analyzed. These varying levels of recombination deep in the tree show that recombination may be playing a different role in the evolution of each group and that broad generalizations to the species as a whole may not apply. Additionally, selection may be playing a role in the differing levels of recombination as some groups may be under more intense selective drug pressure than others. A recent study looking across the core genome of the *S*. *aureus* genome found that homoplasy rates varied on both a broad and fine scale as well [13].

The rates of evolution for all the taxa in this study were greater than rates estimated by other studies [4, 6, 8], however, the 95% CI overlaps previous estimates. The taxa here are diverse across clonal complexes and while there is a demonstrated level of homoplasy due to genomic region swapping across lineages, our analysis filtered those regions from the data set so should not be confounded by recombination. Other studies that estimate substitution rates for *S*. *aureus* included only strains of a single ST, which did not have the same levels of recombination as demonstrated in this study. However, the rate estimates for the individual lineages, CC8 and CC5, were 10^−5^ and 10^−3^, substitutions per nucleotide site per year, respectively. Interestingly, a 10-fold difference in rates between these two groups was found in a study that followed individual patients and sampled repeatedly to determine the microevolutionary SNP accumulation rate over time [50]. However, the significance of this rate difference remains unclear. The TMRCA estimates for CC8 and CC5 indicate that CC5 is a more recently emerged group.

We found evidence of the *S*. *aureus* genome’s flexibility when examining virulence gene distribution. Our results showed differing patterns of virulence gene distribution across separate lineages for some genes and no differences for others, as has been seen in other studies[54, 55]. For example, the capsular polysaccharide genes that define the CP5 and CP8 showed distinct lineage specific variation. Previous studies have shown similar distribution of the *cap5* and *cap8* genes, where each clonal group is dominated by a single capsular type [56, 57]. Additionally, the staphylococcal enterotoxin genes, most of which are not on mobile genetic elements, support groupings based on SNP phylogenies within the CC8 clade. There is a distinct set of the staphylococcal enterotoxin genes absent in the sub-clade that contains the USA300 strains, with only *sek* and *seq* gene products fully present. However, the USA500 group within the same clade had three additional staphylococcal enterotoxin genes present, indicating a loss of some virulence factors in the more derived, but highly successful USA300 group. A similar pattern of staphylococcal enterotoxin genes was noted in an MLST and gene analysis of CC8 strains [48]. Another successful clone, ST398, that is the predominant clone in pigs, shows a similar lack of many virulence genes in our analysis. This could be due to a potential adaptation to non-human hosts associated with the loss of human-related virulence factors shed in the process of adaptation [5]. This flexibility of the *S*. *aureus* genome allows for adaptive radiation of successful lineages, which appears to be the hallmark of this organism. Mobile genetic elements that carry many of the virulence factors in *S*. *aureus* are often lineage associated [58–60]

An examination of our phylogeny of diverse *S*. *aureus* strains highlights some important insights about the relatedness of well-know lineages. The CC8 clade shows a tightly clustered group of USA300 isolates within the CC8 clade containing few SNPs (221 SNPs; 9 parsimony informative SNPs), suggesting a short evolutionary history of this strain that dominates in the US [61–63]. The USA600 (ST45) isolates are distant from all other clades in our analysis; its basal location on the rooted tree signifies an early divergence from other *S*. *aureus*. Our data demonstrates the genetic uniqueness of this group and that its evolution may be distinct from other *S*. *aureus* clades, which could contribute to the apparent increased virulence noted in some strains of this clade [64].

The USA100 isolates, while not a distinct clade, did group separately from the other strains of CC5, notably the USA800s. USA100 has been historically known as a dominant HA-MRSA strain in the USA and is still thought to be the most common strain of nasal MRSA isolates [19, 62]. These genomes also carry a distinct group of virulence genes, as has been reported for HA strains [16, 56, 57], and may indicate separate evolutionary pressures from community-associated (CA) MRSA, even within the same clade.

While ST72 was originally thought to belong to CC8, recent evidence based on MLST data as well as 170 additional distinct genes suggest that this group is the product of recombination between CC5 and CC8 [7]. Our analysis lends further evidence to this hypothesis, given the location of two ST72 (USA700) strains on the phylogeny between the CC8 and CC5 clades.

Comparisons between US strains to those originating in other countries reveal some interesting insights into *S*. *aureus* relatedness across large geographic distances. The two strains that originated in Denmark were previously typed as CC80-MRSA-IV—the most prevalent European MRSA clone [65–68] and grouped close to the CC5 and CC8 clades, indicating that these successful MRSA clades in Europe share genetic history with the successful clades in the US. Future studies will be necessary to identify genetic similarities that lead to successful clone establishment. The ST93 strain, JKD6159, which is the predominant CA-MRSA clone in Australia [69], lies at a significant genetic distance from other *S*. *aureus* lineages in our phylogentic tree. The genome-wide SNP phylogeny supports the previous finding from a comparison of coding sequences showing the highly successful ST93 clone is divergent from other previously sequenced genomes of *S*. *aureus*, particularly other CA strains [70, 71]. Further, the three lineages containing a majority of CA strains, CC30, CC8 and CC80 are distant on the phylogeny, suggesting they don’t share a common ancestor or traits and that these groups have evolved independently, multiple times.

### Limitations

Despite the significant conclusions we can draw from this phylogenetic analysis of *S*. *aureus* strains across diverse types, a sample selection bias likely exists. Our strain selection relied largely on publically available strains representing the PFGE types predominant in the US; however MSSA strains are underrepresented resulting in a MRSA-dominated tree. The results indicate that there is extensive microevolution in the major clades in the US. This is also likely the case in other parts of the world. However, the sample size is less than optimal to make conclusions regarding non-US strains.

## Supporting Information

S1 Fig Phylogenetic tree of strains in study including *S*. *epidermidis*, demonstrating the CC45-USA600 clade is root. Maximum-parsimony tree based on 42,810 SNPs and having a consistency index of 0.66. The numbers on the branches are branch lengths.
(TIFF)

S1 Table Characteristics of *Staphylococcus aureus* isolates used in this study, listed in same order as in phylogeny. (XLS)

S2 Table Virulence genes screened *in silico*. (XLS)

S3 Table PS and SS marginal likelihood estimates for the overall tree, CC5 and CC8. (XLSX)

S4 Table Sequencing and assembly statistics on genomes sequenced in this study. (XLS)

S5 Table Results of BSR in silico gene screen, strains listed in same order as phylogeny. (XLS)

## References

[1] Chambers HF, Deleo FR. Waves of resistance: Staphylococcus aureus in the antibiotic era. Nat Rev Microbiol. 2009;7(9):629–41. Epub 2009/08/15. 10.1038/nrmicro2200 19680247 PMC2871281

[2] Jevons M. “Celbenin” - resistant Staphylococci. Br Med J. 1961;1:124–5. doi: 10.1136/bmj.

[3] Klevens RM, Morrison MA, Nadle J, Petit S, Gershman K, Ray S, et al Invasive methicillin-resistant Staphylococcus aureus infections in the United States. Jama. 2007;298(15):1763–71. Epub 2007/10/18. 10.1001/jama.298.15.1763 . 17940231

[4] Harris SR, Feil EJ, Holden MTG, Quail MA, Nickerson EK, Chantratita N, et al Evolution of MRSA During Hospital Transmission and Intercontinental Spread. Science. 2010;327(5964):469–74. 10.1126/science.1182395 20093474 PMC2821690

[5] Price LB, Stegger M, Hasman H, Aziz M, Larsen J, Andersen PS, et al Staphylococcus aureus CC398: host adaptation and emergence of methicillin resistance in livestock. mBio. 2012;3(1). Epub 2012/02/23. 10.1128/mBio.00305-11 22354957 PMC3280451

[6] Holden MT, Hsu LY, Kurt K, Weinert LA, Mather AE, Harris SR, et al A genomic portrait of the emergence, evolution, and global spread of a methicillin-resistant Staphylococcus aureus pandemic. Genome Res. 2013;23(4):653–64. Epub 2013/01/10. 10.1101/gr.147710.112 23299977 PMC3613582

[7] Monecke S, Coombs G, Shore AC, Coleman DC, Akpaka P, Borg M, et al A field guide to pandemic, epidemic and sporadic clones of methicillin-resistant Staphylococcus aureus. PLoS ONE. 2011;6(4):e17936 Epub 2011/04/16. 10.1371/journal.pone.0017936 21494333 PMC3071808

[8] Nubel U, Dordel J, Kurt K, Strommenger B, Westh H, Shukla SK, et al A timescale for evolution, population expansion, and spatial spread of an emerging clone of methicillin-resistant Staphylococcus aureus. PLos Pathogens. 2010;6(4):e1000855 Epub 2010/04/14. 10.1371/journal.ppat.1000855 20386717 PMC2851736

[9] Kennedy AD, Otto M, Braughton KR, Whitney AR, Chen L, Mathema B, et al Epidemic community-associated methicillin-resistant Staphylococcus aureus: recent clonal expansion and diversification. Proc Natl Acad Sci U S A. 2008;105(4):1327–32. Epub 2008/01/25. 10.1073/pnas.0710217105 18216255 PMC2234137

[10] Sanguinetti L, Toti S, Reguzzi V, Bagnoli F, Donati C. A novel computational method identifies intra- and inter-species recombination events in Staphylococcus aureus and Streptococcus pneumoniae. PLoS Comput Biol. 2012;8(9):e1002668 Epub 2012/09/13. 10.1371/journal.pcbi.1002668 22969418 PMC3435249

[11] Feil EJ, Cooper JE, Grundmann H, Robinson DA, Enright MC, Berendt T, et al How clonal is Staphylococcus aureus? Journal of Bacteriology. 2003;185(11):3307–16. Epub 2003/05/20. 12754228 10.1128/JB.185.11.3307-3316.2003 PMC155367

[12] Takuno S, Kado T, Sugino RP, Nakhleh L, Innan H. Population genomics in bacteria: a case study of Staphylococcus aureus. Molecular Biology and Evolution. 2012;29(2):797–809. Epub 2011/10/20. 10.1093/molbev/msr249 22009061 PMC3350317

[13] Everitt RG, Didelot X, Batty EM, Miller RR, Knox K, Young BC, et al Mobile elements drive recombination hotspots in the core genome of Staphylococcus aureus. Nature communications. 2014;5:3956 10.1038/ncomms4956 24853639 PMC4036114

[14] Diep BA, Otto M. The role of virulence determinants in community-associated MRSA pathogenesis. Trends Microbiol. 2008;16(8):361–9. Epub 2008/07/01. 10.1016/j.tim.2008.05.002 18585915 PMC2778837

[15] Varshney AK, Mediavilla JR, Robiou N, Guh A, Wang X, Gialanella P, et al Diverse enterotoxin gene profiles among clonal complexes of Staphylococcus aureus isolates from the Bronx, New York. Appl Environ Microbiol. 2009;75(21):6839–49. Epub 2009/09/15. 10.1128/AEM.00272-09 19749060 PMC2772442

[16] Diep BA, Carleton HA, Chang RF, Sensabaugh GF, Perdreau-Remington F. Roles of 34 virulence genes in the evolution of hospital- and community-associated strains of methicillin-resistant Staphylococcus aureus. The Journal of Infectious Diseases. 2006;193(11):1495–503. Epub 2006/05/03. 10.1086/503777 . 16652276

[17] Sahl JW, Gillece JD, Schupp JM, Waddell VG, Driebe EM, Engelthaler DM, et al Evolution of a pathogen: a comparative genomics analysis identifies a genetic pathway to pathogenesis in acinetobacter. PLoS ONE. 2013;8(1):e54287 Epub 2013/02/01. 10.1371/journal.pone.0054287 . 23365658 PMC3554770

[18] Tenover FC, Gay EA, Frye S, Eells SJ, Healy M, McGowan JE Jr. Comparison of typing results obtained for methicillin-resistant Staphylococcus aureus isolates with the DiversiLab system and pulsed-field gel electrophoresis. Journal of Clinical Microbiology. 2009;47(8):2452–7. Epub 2009/06/26. 10.1128/JCM.00476-09 19553588 PMC2725641

[19] McDougal LK, Steward CD, Killgore GE, Chaitram JM, McAllister SK, Tenover FC. Pulsed-Field Gel Electrophoresis Typing of Oxacillin-Resistant Staphylococcus aureus Isolates from the United States: Establishing a National Database. Journal of Clinical Microbiology. 2003;41(11):5113–20. 10.1128/jcm.41.11.5113-5120.2003 14605147 PMC262524

[20] Zerbino DR, Birney E. Velvet: algorithms for de novo short read assembly using de Bruijn graphs. Genome Res. 2008;18(5):821–9. Epub 2008/03/20. 10.1101/gr.074492.107 18349386 PMC2336801

[21] Kurtz S, Phillippy A, Delcher AL, Smoot M, Shumway M, Antonescu C, et al Versatile and open software for comparing large genomes. Genome Biol. 2004;5(2):R12 Epub 2004/02/05. 10.1186/gb-2004-5-2-r12 14759262 PMC395750

[22] Tamura K, Dudley J, Nei M, Kumar S. MEGA4: Molecular Evolutionary Genetics Analysis (MEGA) software version 4.0. Molecular Biology and Evolution. 2007;24(8):1596–9. Epub 2007/05/10. 10.1093/molbev/msm092 . 17488738

[23] Tamura K, Peterson D, Peterson N, Stecher G, Nei M, Kumar S. MEGA5: molecular evolutionary genetics analysis using maximum likelihood, evolutionary distance, and maximum parsimony methods. Molecular Biology and Evolution. 2011;28(10):2731–9. Epub 2011/05/07. 10.1093/molbev/msr121 21546353 PMC3203626

[24] Larsen AR, Stegger M, Sorum M. spa typing directly from a mecA, spa and pvl multiplex PCR assay-a cost-effective improvement for methicillin-resistant Staphylococcus aureus surveillance. Clin Microbiol Infect. 2008;14(6):611–4. Epub 2008/04/09. 10.1111/j.1469-0691.2008.01995.x . 18393997

[25] Rasko DA, Myers GS, Ravel J. Visualization of comparative genomic analyses by BLAST score ratio. BMC Bioinformatics. 2005;6:2 Epub 2005/01/07. 10.1186/1471-2105-6-2 15634352 PMC545078

[26] Altschul SF, Madden TL, Schaffer AA, Zhang J, Zhang Z, Miller W, et al Gapped BLAST and PSI-BLAST: a new generation of protein database search programs. Nucleic Acids Res. 1997;25(17):3389–402. Epub 1997/09/01. 9254694 10.1093/nar/25.17.3389 PMC146917

[27] Saeed AI, Bhagabati NK, Braisted JC, Liang W, Sharov V, Howe EA, et al TM4 microarray software suite. Methods Enzymol. 2006;411:134–93. Epub 2006/08/31. S0076-6879(06)11009-5 [pii] 10.1016/S0076-6879(06)11009-5 . 16939790

[28] Diep BA, Gill SR, Chang RF, Phan TH, Chen JH, Davidson MG, et al Complete genome sequence of USA300, an epidemic clone of community-acquired meticillin-resistant Staphylococcus aureus. Lancet. 2006;367(9512):731–9. Epub 2006/03/07. 10.1016/S0140-6736(06)68231-7 . 16517273

[29] Kuroda M, Ohta T, Uchiyama I, Baba T, Yuzawa H, Kobayashi I, et al Whole genome sequencing of meticillin-resistant Staphylococcus aureus. Lancet. 2001;357(9264):1225–40. Epub 2001/06/22. . 11418146 10.1016/s0140-6736(00)04403-2

[30] Homer N, Merriman B, Nelson SF. BFAST: an alignment tool for large scale genome resequencing. PLoS ONE. 2009;4(11):e7767 Epub 2009/11/13. 10.1371/journal.pone.0007767 19907642 PMC2770639

[31] Pearson T, Hornstra HM, Sahl JW, Schaack S, Schupp JM, Beckstrom-Sternberg SM, et al. When Outgroups Fail; Phylogenomics of Rooting the Emerging Pathogen, Coxiella burnetii. Systematic biology. 2013. Epub 2013/06/06. 10.1093/sysbio/syt038 . 23736103 PMC3739886

[32] Sanderson MJ, Hufford L. Homoplasy: the recurrence of similarity in evolution San Diego: Academic Press; 1996. xxv, 339 p. p.

[33] Hasegawa M, Kishino H, Yano T. Dating of the human-ape splitting by a molecular clock of mitochondrial DNA. Journal of molecular evolution. 1985;22(2):160–74. 10.1007/BF02101694 . 3934395

[34] Krzywinski M, Schein J, Birol I, Connors J, Gascoyne R, Horsman D, et al Circos: an information aesthetic for comparative genomics. Genome Res. 2009;19(9):1639–45. Epub 2009/06/23. 10.1101/gr.092759.109 19541911 PMC2752132

[35] Drummond AJ, Suchard MA, Xie D, Rambaut A. Bayesian phylogenetics with BEAUti and the BEAST 1.7. Mol Biol Evol. 2012;29(8):1969–73. 10.1093/molbev/mss075 22367748 PMC3408070

[36] Gray RR, Tatem AJ, Johnson JA, Alekseyenko AV, Pybus OG, Suchard MA, et al Testing Spatiotemporal Hypothesis of Bacterial Evolution Using Methicillin-Resistant Staphylococcus aureus ST239 Genome-wide Data within a Bayesian Framework. Molecular Biology and Evolution. 2010;28(5):1593–603. 10.1093/molbev/msq319 21112962 PMC3115679

[37] Gelman AM, Xiao-Li. Simulating normalizing constants: from importance sampling to bridge sampling to path sampling. Statistical Science. 1998;13(2):163–85. 10.1214/ss/1028905934

[38] Xie W, Lewis PO, Fan Y, Kuo L, Chen MH. Improving marginal likelihood estimation for Bayesian phylogenetic model selection. Systematic biology. 2011;60(2):150–60. 10.1093/sysbio/syq085 21187451 PMC3038348

[39] Baele G, Lemey P, Bedford T, Rambaut A, Suchard MA, Alekseyenko AV. Improving the accuracy of demographic and molecular clock model comparison while accommodating phylogenetic uncertainty. Mol Biol Evol. 2012;29(9):2157–67. 10.1093/molbev/mss084 22403239 PMC3424409

[40] Baele G, Li WL, Drummond AJ, Suchard MA, Lemey P. Accurate model selection of relaxed molecular clocks in bayesian phylogenetics. Mol Biol Evol. 2013;30(2):239–43. 10.1093/molbev/mss243 23090976 PMC3548314

[41] Drummond AJ, Ho SY, Phillips MJ, Rambaut A. Relaxed phylogenetics and dating with confidence. PLoS biology. 2006;4(5):e88 10.1371/journal.pbio.0040088 16683862 PMC1395354

[42] Faria NR, Rambaut A, Suchard MA, Baele G, Bedford T, Ward MJ, et al HIV epidemiology. The early spread and epidemic ignition of HIV-1 in human populations. Science. 2014;346(6205):56–61. 10.1126/science.1256739 25278604 PMC4254776

[43] Rambaut A SM, Xie D & Drummond AJ. Tracer v1.6. 2014. Available: http://beast.bio.ed.ac.uk/Tracer.

[44] Holt DC, Holden MT, Tong SY, Castillo-Ramirez S, Clarke L, Quail MA, et al A very early-branching Staphylococcus aureus lineage lacking the carotenoid pigment staphyloxanthin. Genome Biol Evol. 2011;3:881–95. Epub 2011/08/05. 10.1093/gbe/evr078 21813488 PMC3175761

[45] Robinson DA, Enright MC. Evolution of Staphylococcus aureus by Large Chromosomal Replacements. Journal of Bacteriology. 2004;186(4):1060–4. 10.1128/jb.186.4.1060-1064.2004 14762000 PMC344219

[46] Holden MT, Lindsay JA, Corton C, Quail MA, Cockfield JD, Pathak S, et al Genome sequence of a recently emerged, highly transmissible, multi-antibiotic- and antiseptic-resistant variant of methicillin-resistant Staphylococcus aureus, sequence type 239 (TW). J Bacteriol. 2010;192(3):888–92. 10.1128/JB.01255-09 19948800 PMC2812470

[47] Nubel U, Roumagnac P, Feldkamp M, Song JH, Ko KS, Huang YC, et al Frequent emergence and limited geographic dispersal of methicillin-resistant Staphylococcus aureus. Proc Natl Acad Sci U S A. 2008;105(37):14130–5. Epub 2008/09/06. 10.1073/pnas.0804178105 18772392 PMC2544590

[48] Li M, Diep BA, Villaruz AE, Braughton KR, Jiang X, DeLeo FR, et al Evolution of virulence in epidemic community-associated methicillin-resistant Staphylococcus aureus. Proc Natl Acad Sci U S A. 2009;106(14):5883–8. Epub 2009/03/19. 10.1073/pnas.0900743106 19293374 PMC2667066

[49] Gorwitz RJ, Kruszon‐Moran D, McAllister SK, McQuillan G, McDougal LK, Fosheim GE, et al Changes in the Prevalence of Nasal Colonization withStaphylococcus aureusin the United States, 2001–2004. The Journal of Infectious Diseases. 2008;197(9):1226–34. 10.1086/533494 18422434

[50] Long SW, Beres SB, Olsen RJ, Musser JM. Absence of patient-to-patient intrahospital transmission of Staphylococcus aureus as determined by whole-genome sequencing. MBio. 2014;5(5):e01692–14. 10.1128/mBio.01692-14 25293757 PMC4196229

[51] Voyich JM, Otto M, Mathema B, Braughton KR, Whitney AR, Welty D, et al Is Panton-Valentine leukocidin the major virulence determinant in community-associated methicillin-resistant Staphylococcus aureus disease? The Journal of Infectious Diseases. 2006;194(12):1761–70. Epub 2006/11/17. 10.1086/509506 . 17109350

[52] Bae IG, Tonthat GT, Stryjewski ME, Rude TH, Reilly LF, Barriere SL, et al Presence of genes encoding the panton-valentine leukocidin exotoxin is not the primary determinant of outcome in patients with complicated skin and skin structure infections due to methicillin-resistant Staphylococcus aureus: results of a multinational trial. Journal of Clinical Microbiology. 2009;47(12):3952–7. Epub 2009/10/23. 10.1128/JCM.01643-09 19846653 PMC2786648

[53] Vandenesch F, Naimi T, Enright MC, Lina G, Nimmo GR, Heffernan H, et al Community-acquired methicillin-resistant Staphylococcus aureus carrying Panton-Valentine leukocidin genes: worldwide emergence. Emerging Infectious Diseases. 2003;9(8):978–84. Epub 2003/09/12. 10.3201/eid0908.030089 12967497 PMC3020611

[54] Lindsay JA, Holden MT. Understanding the rise of the superbug: investigation of the evolution and genomic variation of Staphylococcus aureus. Functional & integrative genomics. 2006;6(3):186–201. 10.1007/s10142-005-0019-7 . 16453141

[55] Sung JM, Lindsay JA. Staphylococcus aureus strains that are hypersusceptible to resistance gene transfer from enterococci. Antimicrob Agents Chemother. 2007;51(6):2189–91. 10.1128/AAC.01442-06 17371826 PMC1891402

[56] Lattar SM, Tuchscherr LP, Centron D, Becker K, Predari SC, Buzzola FR, et al Molecular fingerprinting of Staphylococcus aureus isolated from patients with osteomyelitis in Argentina and clonal distribution of the cap5(8) genes and of other selected virulence genes. Eur J Clin Microbiol Infect Dis. 2012;31(10):2559–66. Epub 2012/03/28. 10.1007/s10096-012-1596-8 22450741 PMC3422409

[57] Moore PC, Lindsay JA. Genetic variation among hospital isolates of methicillin-sensitive Staphylococcus aureus: evidence for horizontal transfer of virulence genes. Journal of Clinical Microbiology. 2001;39(8):2760–7. Epub 2001/07/28. 10.1128/JCM.39.8.2760-2767.2001 11473989 PMC88236

[58] Waldron DE, Lindsay JA. Sau1: a novel lineage-specific type I restriction-modification system that blocks horizontal gene transfer into Staphylococcus aureus and between S. aureus isolates of different lineages. J Bacteriol. 2006;188(15):5578–85. 10.1128/JB.00418-06 16855248 PMC1540015

[59] McCarthy AJ, Lindsay JA. The distribution of plasmids that carry virulence and resistance genes in Staphylococcus aureus is lineage associated. BMC Microbiol. 2012;12:104 10.1186/1471-2180-12-104 22691167 PMC3406946

[60] McCarthy AJ, Witney AA, Lindsay JA. Staphylococcus aureus temperate bacteriophage: carriage and horizontal gene transfer is lineage associated. Frontiers in cellular and infection microbiology. 2012;2:6 10.3389/fcimb.2012.00006 22919598 PMC3417521

[61] Pasquale TR, Jabrocki B, Salstrom SJ, Wiemken TL, Peyrani P, Haque NZ, et al. Emergence of methicillin-resistant Staphylococcus aureus USA300 genotype as a major cause of late-onset nosocomial pneumonia in intensive care patients in the USA. Int J Infect Dis. 2013. Epub 2013/02/05. 10.1016/j.ijid.2012.12.013 . 23375542

[62] Tenover FC, Tickler IA, Goering RV, Kreiswirth BN, Mediavilla JR, Persing DH. Characterization of Nasal and Blood Culture Isolates of Methicillin-Resistant Staphylococcus aureus from Patients in United States Hospitals. Antimicrobial Agents and Chemotherapy. 2011;56(3):1324–30. 10.1128/aac.05804-11 22155818 PMC3294931

[63] Jackson CR, Davis JA, Barrett JB. Prevalence and characterization of Methicillin-resistant Staphylococcus aureus isolated from retail meat and humans in Georgia. Journal of Clinical Microbiology. 2013. Epub 2013/02/01. 10.1128/JCM.03166-12 . 23363837 PMC3666775

[64] Moore CL, Osaki-Kiyan P, Perri M, Donabedian S, Haque NZ, Chen A, et al USA600 (ST45) Methicillin-Resistant Staphylococcus aureus Bloodstream Infections in Urban Detroit. Journal of Clinical Microbiology. 2010;48(6):2307–10. 10.1128/jcm.00409-10 20335422 PMC2884493

[65] Hanssen AM, Fossum A, Mikalsen J, Halvorsen DS, Bukholm G, Sollid JU. Dissemination of community-acquired methicillin-resistant Staphylococcus aureus clones in northern Norway: sequence types 8 and 80 predominate. J Clin Microbiol. 2005;43(5):2118–24. 10.1128/JCM.43.5.2118-2124.2005 15872230 PMC1153739

[66] Stam-Bolink EM, Mithoe D, Baas WH, Arends JP, Moller AV. Spread of a methicillin-resistant Staphylococcus aureus ST80 strain in the community of the northern Netherlands. Eur J Clin Microbiol Infect Dis. 2007;26(10):723–7. 10.1007/s10096-007-0352-y 17636366 PMC2039805

[67] Witte W, Braulke C, Cuny C, Strommenger B, Werner G, Heuck D, et al Emergence of methicillin-resistant Staphylococcus aureus with Panton-Valentine leukocidin genes in central Europe. Eur J Clin Microbiol Infect Dis. 2005;24(1):1–5. 10.1007/s10096-004-1262-x . 15599784

[68] Brauner J, Hallin M, Deplano A, De Mendonca R, Nonhoff C, De Ryck R, et al Community-acquired methicillin-resistant Staphylococcus aureus clones circulating in Belgium from 2005 to 2009: changing epidemiology. Eur J Clin Microbiol Infect Dis. 2013;32(5):613–20. 10.1007/s10096-012-1784-6 . 23232976

[69] Coombs GW, Nimmo GR, Pearson JC, Christiansen KJ, Bell JM, Collignon PJ, et al Prevalence of MRSA strains among Staphylococcus aureus isolated from outpatients, 2006. Commun Dis Intell. 2009;33(1):10–20. Epub 2009/07/22. . 19618763 10.33321/cdi.2009.33.2

[70] Chua K, Seemann T, Harrison PF, Davies JK, Coutts SJ, Chen H, et al Complete genome sequence of Staphylococcus aureus strain JKD6159, a unique Australian clone of ST93-IV community methicillin-resistant Staphylococcus aureus. Journal of Bacteriology. 2010;192(20):5556–7. Epub 2010/08/24. 10.1128/JB.00878-10 20729356 PMC2950503

[71] Chua KY, Seemann T, Harrison PF, Monagle S, Korman TM, Johnson PD, et al The dominant Australian community-acquired methicillin-resistant Staphylococcus aureus clone ST93-IV [2B] is highly virulent and genetically distinct. PLoS ONE. 2011;6(10):e25887 Epub 2011/10/13. 10.1371/journal.pone.0025887 21991381 PMC3185049

