# Supplementary figures and images for: Using Whole Genome Analysis to Examine Recombination across Diverse Sequence Types of Staphylococcus aureus
Source: PLoS One. 2015 Jul 10;10(7):e0130955. doi: 10.1371/journal.pone.0130955 (PMC4498916; doi:10.1371/journal.pone.0130955)

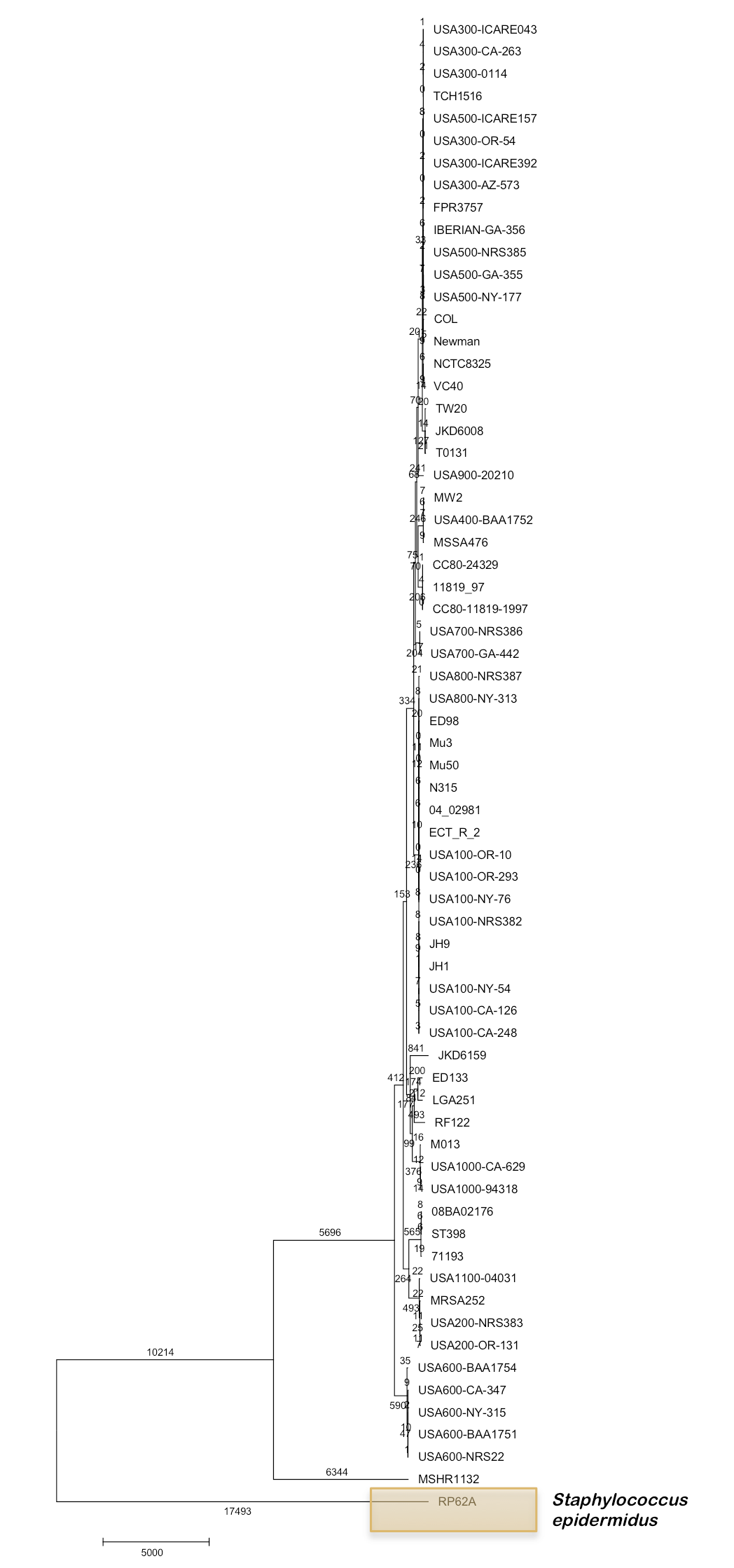

Supplement: S1 Fig — Maximum-parsimony tree based on 42,810 SNPs and having a consistency index of 0.66. The numbers on the branches are branch lengths. (TIFF) [file pone.0130955.s001.tiff]
